# Functional Trait Strategies of Trees in Dry and Wet Tropical Forests Are Similar but Differ in Their Consequences for Succession

**DOI:** 10.1371/journal.pone.0123741

**Published:** 2015-04-28

**Authors:** Madelon Lohbeck, Edwin Lebrija-Trejos, Miguel Martínez-Ramos, Jorge A. Meave, Lourens Poorter, Frans Bongers

**Affiliations:** 1 Forest Ecology and Forest Management Group, Wageningen University, PO Box 47, 6700 AA, Wageningen, The Netherlands; 2 Smithsonian Tropical Research Institute, Apartado 0843-03092, Balboa, Ancon, Panama; 3 Centro de Investigaciones en Ecosistemas, Universidad Nacional Autónoma de México, Campus Morelia, Antigua Carretera a Pátzcuaro 8701, Ex-hacienda de San José de la Huerta, 58190, Morelia, Michoacán, Mexico; 4 Departamento de Ecología y Recursos Naturales, Facultad de Ciencias, Universidad Nacional Autónoma de México, 04510, México, Distrito Federal, Mexico; Berkeley, UNITED STATES

## Abstract

Global plant trait studies have revealed fundamental trade-offs in plant resource economics. We evaluated such trait trade-offs during secondary succession in two species-rich tropical ecosystems that contrast in precipitation: dry deciduous and wet evergreen forests of Mexico. Species turnover with succession in dry forest largely relates to increasing water availability and in wet forest to decreasing light availability. We hypothesized that while functional trait trade-offs are similar in the two forest systems, the successful plant strategies in these communities will be different, as contrasting filters affect species turnover. Research was carried out in 15 dry secondary forest sites (5-63 years after abandonment) and in 17 wet secondary forest sites (<1-25 years after abandonment). We used 11 functional traits measured on 132 species to make species-trait PCA biplots for dry and wet forest and compare trait trade-offs. We evaluated whether multivariate plant strategies changed during succession, by calculating a ‘Community-Weighted Mean’ plant strategy, based on species scores on the first two PCA-axes. Trait spectra reflected two main trade-off axes that were similar for dry and wet forest species: acquisitive versus conservative species, and drought avoiding species versus evergreen species with large animal-dispersed seeds. These trait associations were consistent when accounting for evolutionary history. Successional changes in the most successful plant strategies reflected different functional trait spectra depending on the forest type. In dry forest the community changed from having drought avoiding strategies early in succession to increased abundance of evergreen strategies with larger seeds late in succession. In wet forest the community changed from species having mainly acquisitive strategies to those with more conservative strategies during succession. These strategy changes were explained by increasing water availability during dry forest succession and increasing light scarcity during wet forest succession. Although similar trait spectra were observed among dry and wet secondary forest species, the consequences for succession were different resulting from contrasting environmental filters.

## Introduction

Trade-offs in plant traits and resource economics are consistent at the global scale [1–3]. These give insight into comprehensive dimensions of multivariate functional trait variation, or what we call ‘functional trait spectra’. As functional traits are indicators of ecological strategies, the study of trait spectra and trade-offs allows us to explore the complex interplay of different strategies [4]. For example, the worldwide leaf economics spectrum runs from a plant strategy with cheap-to-construct acquisitive leaves with high photosynthetic rates that maximize resource capture to a strategy with expensive-to-construct conservative leaves that tolerate stress and physical damage and better conserve the acquired resources [2]. Such an economic spectrum has not only been found for leaves, but also for other plant organs like roots and stems [3,5], and it has been found across different climatic regions [2,6]. This economics spectrum at the tissue level underlies the trade-off between growth and survival at the whole-plant level [7], as in resource rich environments acquisitive strategies thrive by fast growth (and high mortality) whilst in resource-poor environments conservative strategies thrive by persistence (and high survival). This fundamental trade-off describes variation among plants in the established phase. In contrast, different trade-offs are found in the regenerative phase, where plants have to arrive and establish successfully at a site. As a result, traits related to the regenerative phase are largely decoupled from those related to the established phase [8]. The trade-off between seed size and seed number plays an important role in explaining the differential success of species in the regenerative phase, e.g. [9]. Small seeds are produced in large numbers and are often wind-dispersed, which is advantageous when colonizing new sites [10], but their small seed size comes at the expense of a lower per capita establishment success [11]. Large seeds produce robust seedlings [12], which is advantageous when colonizing shaded sites [13], and they are often animal-dispersed, enhancing directed dispersal to safe sites [14].

These traits and trait trade-offs are used to explain species’ success along successional gradients. In tropical wet forest, succession is related to a gradient of decreasing light availability over time, e.g. [15], whereas in dry forest it is related to a gradient of increasing water availability over time [16,17]. Dry forest species experience, therefore, stressful conditions during the dry and hot early stages of succession, while wet forest species do so during the shaded late stages of succession. We showed previously that, at the *community-level*, the community-weighted mean (CWM) of *individual* functional traits changed with tropical forest succession in Mexico [17]. The type of traits that changed differed largely between dry and wet forests [18]. In dry forest early-successional communities had trait values related to drought tolerance and optimal light acquisition, whereas late-successional communities had trait values related to large seeds and biotic dispersal. In wet forest early-successional communities also had trait values related to optimal light acquisition, whereas late-successional communities had trait values related to increased leaf toughness. Here we expand on the previous analysis, and explore differences in *species-level* trait trade-offs between dry and wet forest species, and to what extent this can be translated into different *multivariate plant strategies* between dry and wet forest species. Since environmental gradients filter species based on multiple traits, identifying changes in multivariate plant strategies is needed to further advance our understanding of ecological restoration, cf. [19].

The present study focuses on trait trade-offs *at the species level*, and how *multivariate plant strategies* change during succession. To this end we described plant strategies using 11 functional traits measured on 132 species found in 32 secondary forest sites belonging to dry and wet tropical forest in Mexico. We hypothesized the existence of two major trait- or strategy spectra, namely the spectrum of species with acquisitive versus those with conservative trait values, which is important in the established phase of plants, and the spectrum of small seeded wind-dispersed species versus large seeded animal-dispersed species, which is important in the regeneration phase. We expected that in dry forest water is the main limiting factor, and that tree communities show a change from predominantly conservative to acquisitive strategies over time, whereas in wet forest light is the main limiting factor, and the communities show a change from predominantly acquisitive to conservative strategies over time. We also expected that the seed size spectrum would play an important role in both forest types, reflecting an increase in the proportion of large seeded animal-dispersed species along succession.

## Methods

### Ethics statement

Since all secondary forest plots are located on privately owned land, permission from landowners to enter the sites and collect plant material was provided before conducting this research.

### Research locations

#### Tropical dry forest

Research plots in tropical dry forest surround the village of Nizanda on the Pacific watershed of the Isthmus of Tehuantepec in Oaxaca, southern Mexico (16°39’N, 95°00’W). Mean annual temperature is 26°C and mean annual precipitation is 900 mm, of which > 90% concentrates between late May and mid-October [20]. The vegetation is predominantly tropical dry deciduous forest, characterized by a low canopy stature (ca. 7 m tall) [21,22]. The 15 secondary forest plots (900 m^2^) with different fallow ages (6–64 years) were established on abandoned maize fields. Within each plot four parallel 5 × 20 m transects were set up, and further divided into four 5 × 5 m quadrats. In one quadrat all individuals with DBH ≥ 1cm were identified and measured, in a second all individuals with DBH ≥ 2.5 cm and in the remaining two all individuals with DBH ≥ 5 cm, with these sampling criteria being randomly assigned to each quadrat. Variables measured on each individual were scaled up to the plot level according to sampling effort per size-class (i.e., all stems 1 cm ≤ DBH ≤ 2.5 cm were multiplied by four, and 2.5 cm ≤ DBH ≤ 5 cm by two, to make sampling effort comparable across size-classes, after which all stems are added up). For further details see Lebrija-Trejos et al. [20].

#### Tropical wet forest

Research plots in the tropical wet forest surround the village of Loma Bonita in the Marqués de Comillas region in Chiapas, southeastern Mexico (16°01’N, 90°55’W). Mean annual temperature is 24°C and mean annual precipitation is 3000 mm, with a dry period (< 100 mm month^-1^) from February through April [23]. The research area is characterized by small hills and valleys with sandy and clay soils of low pH (< 5.5). The 17 secondary forest plots (1000 m^2^) with different fallow ages (< 1–25 years) were established on abandoned maize fields. Each plot was divided into two 10 × 50 m subplots. In one subplot all individuals with DBH ≥ 1 cm were identified and measured, in the second all individuals DBH ≥ 5 cm. Again, measured variables were scaled to the plot level up according to sampling effort per size-class (i.e., stems 1 cm ≤ DBH ≤ 5 cm were multiplied by two to make sampling effort comparable to DBH ≥ 5 cm, after which all stems are added up).

### Functional traits

Those species that made up at least 80% of the basal area in the plots were selected for functional trait measurements (excluding cacti in dry forest, as their functional traits are difficult to compare with trees), because they accurately describe the community-weighted mean [24,25]. This resulted in a total of 132 species: 51 dry forest species and 81 wet forest species (see S1 Table for the list of species per forest type). We measured seven leaf traits: leaf area (m^2^), specific leaf area- SLA (m^2^/kg), leaf dry matter content- LDMC (g/g), leaf density (g/cm^3^), leaf thickness (mm), leaf compoundness (0 = simple, 1 = compound), petiole length (cm); one whole plant trait: deciduousness (0 = evergreen, 1 = deciduous); one stem trait: wood density- WD (g/cm^3^); and two regenerative traits: seed size (mm^3^) and dispersal syndrome (0 = abiotic, 1 = biotic). Traits were measured following standardized protocols [26,27]. In the wet forest sites, leaf traits were measured for two sun-lit leaves for 10 adult trees per species (5 individuals for specific force to punch) of ca. 5 m high, and in dry forest for 5 sun-lit leaves for 5 adult trees per species with a DBH of 10–30 cm. Functional trait measurements took place within the study areas, but not inside the plots. For wood density measurements in the wet forest 15 of the 81 species were taken from comparable Mexican ecosystems. The binary traits leaf compoundness, deciduousness, and dispersal syndrome were scored based on field observations, local informants, herbaria, and literature; for detailed methods on functional trait measurements see supplementary material in [18]. We used species’ average trait values although we recognize that intraspecific trait variation may play an important role in species adaptation along environmental gradients. However, given the extensive species-level trait data set (132 species) together with the high species turnover during succession, for the purpose of this study we consider the use of species average trait values appropriate to test our hypotheses.

### Statistical analysis

We used principal component analysis to quantify spectra of trait-based multivariate plant strategies for each forest type separately. The PCA biplots show the main trade-offs across (standardized) functional traits based on principal axes of variation, where binary variables are treated as dummy variables. Trait spectra for dry and wet forest species were compared by correlating the correlation coefficients of all pairwise trait combinations; in each site 11 traits were measured, resulting in 55 pairwise trait correlations per site. Subsequently the pairwise trait correlation coefficients derived from dry forest species were correlated with the pairwise trait correlation coefficients derived from wet forest species. Spearman correlation coefficients were used, since not all traits are normally distributed, except for relating the binary variables [deciduousness (De), leaf compoundness (LC) and biotic dispersal (Di)] when we used the Phi coefficient, a measure of association between binary variables whose interpretation is similar to correlation coefficients.

We also examined whether the trait associations found were influenced by evolutionary histories. To this end, we recovered phylogenetic trees for the dry forest species and the wet forest species using Phylomatic [28], scaling branch lengths to one. For all traits and each forest type we explored phylogenetic signal (Blomberg’s K [29]) and compared this to random trait distributions over the phylogenetic tree, using the package “Picante” [30]. Phylogenetically independent contrasts were computed as the difference in the mean trait values for pairs of sister species and nodes, using the package “Ape” [31] and we compared whether trait associations were similar with and without considering phylogeny [32].

Species scores on the first two principal components of the PCA were scaled up to community level using the Community Weighted Mean (CWM) [24,33], which is calculated as follows:

$$\text{CWM}=\;\overset{S}{\underset{i=1}{\sum }}w_{i}\times \;x_{i}$$

where S is the total number of species, *w*_*i*_ is the relative basal area of the i^th^ species and *x*_*i*_ is the score on the PCA axis of the i^th^ species. Relative basal area is a measure of species’ relative contributions to the total basal area represented by functional trait measurements in each plot (which is in turn at least 80% of total basal area in a plot). The relative basal area was used for weighting, rather than the abundance, because it reflects the species’ biomass, an indicator of plant performance and adaptation to local conditions. These community weighted mean scores on the PCA axes reflect the average multivariate plant strategy in the community, and were regressed against stand basal area (m^2^/ha) (including cacti in the case of dry forest). Stand basal area is a structural variable of succession and logarithmically relates to forest age in both forest types [see supplementary material in 18]. Stand basal area was used, and not age, because it better reflects aboveground biomass, understory light interception and environmental conditions [16] as well as competitive interactions [34]. All statistical analyses were carried out using R v. 2.13.1 [35]; for multivariate analysis we used the package ‘Vegan’ [36].

## Results

**Fig 1:**
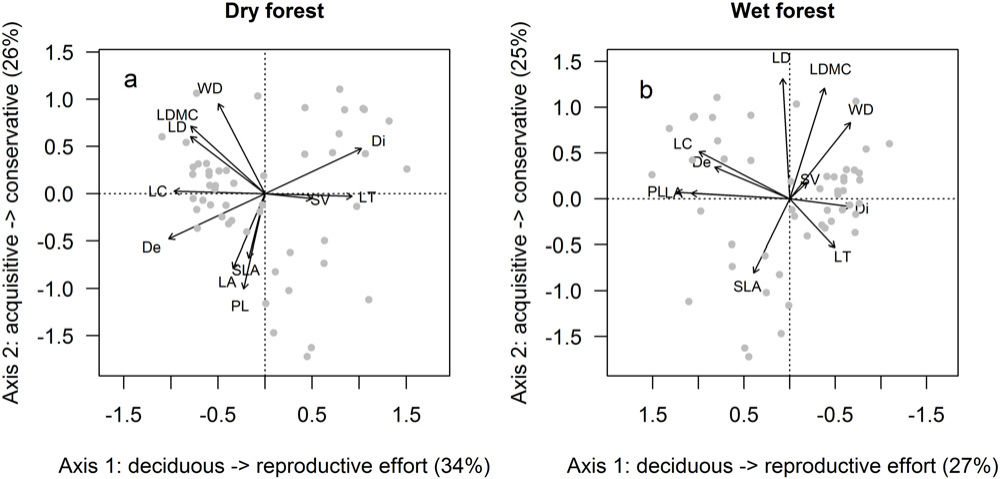
Results of the Principal Component Analyses applied to functional traits of tree species from Mexican tropical dry and wet forests. (a) PCA of dry forest species (n = 51), (b) PCA of wet forest species (n = 81). Species (grey symbols) were separated based on their functional traits shown as arrows; LA = leaf area, SLA = specific leaf area, LD = leaf density, LT = leaf thickness, LDMC = leaf dry matter content, PL = petiole length, WD = wood density, LC = leaf compoundness (0 = simple, 1 = compound), Di = dispersal syndrome (0 = abiotic, 1 = biotic), De = deciduousness (0 = evergreen, 1 = deciduous). LA and PL were ln-transformed.

**Fig 2:**
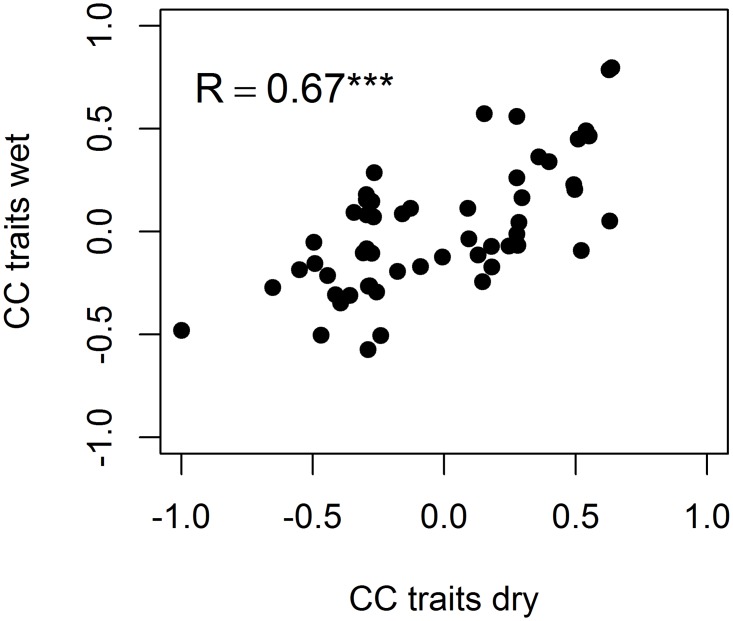
Correlation coefficients (CC) of all pairwise trait combinations (11 traits, resulting in 55 pairwise trait combinations per forest type, see Table 2) of dry forest species plotted against those of wet forest species. Correlation coefficients represent Spearman coefficients except when relating binary variables, then the Phi coefficient was used. The pairwise correlation coefficients of dry forest proved to be significantly correlated with those of the wet forest (Pearson product moment correlation [R], P < 0.001), indicating that trait spectra are consistent across the two different forest types.

The first two component axes of the PCAs for dry and wet forest species captured more than half of the variation in species trait values (Fig 1, Table 1). The ordination biplots indicated that the spectra of functional trait-based strategies of the dry forest species were similar to those of the wet forest species. This was confirmed when the pairwise correlation coefficients of the dry forest were plotted against those of the wet forest (Fig 2, Table 2); the highly significant positive correlation indicated that the same trait associations were found for the species of the two forest types. The first PCA axes were largely related to phenology and reproductive strategies, with deciduous, small-seeded wind-dispersed species on the left side, and species with large seeds, biotic seed dispersal, and thick leaves on the right side (Fig 1). We will therefore refer to this axis as the deciduousness/ reproductive effort strategy axis. The second PCA axes were related to the plant economics spectrum, with species having acquisitive trait values (e.g., high SLA) at the lower side, and those having conservative trait values (e.g., high leaf density, LDMC and WD) at the upper side (Fig 1). We refer to this axis as the acquisitive/conservative strategy axis.

**Table 1:** Eigenvector scores of functional traits on the two main principal components for dry forest and for wet forest.

Traits	Dry forest		Wet forest	
	PC1 (34%)	PC2 (26%)	PC1 (27%)	PC2 (25%)
LA^§^	-0.141	-0.378	0.446	0.026
SLA	-0.072	-0.327	0.163	-0.352
LDMC	-0.327	0.343	-0.158	0.528
LD	-0.328	0.290	0.032	0.574
LT	0.383	-0.014	-0.205	-0.231
PL^§^	-0.094	-0.482	0.513	0.031
LC	-0.401	0.012	0.411	0.225
WD	-0.205	0.458	-0.275	0.364
De	-0.424	-0.230	0.340	0.150
Di	0.424	0.230	-0.279	-0.038
SV	0.206	-0.025	-0.079	0.079

Values in parentheses indicate variance accounted for by each axis.

^§^Variable was ln-transformed.

**Table 2:** Spearman coefficients of the pairwise relations between variables and the principal components (Fig 1).

	PCA1	PCA2	LA^*§*^	SLA	LDMC	LD	LT	PL^*§*^	LC	WD	De	Di	SV
PCA1		-0.08	0.75 ***	0.33 **	-0.33 **	-0.04	-0.36 ***	0.89 ***	0.68 ***	-0.48 ***	0.48 ***	-0.45 ***	-0.23 *
PCA2	-0.01		0.08	-0.59 ***	0.86 ***	0.94 ***	-0.25 *	0.04	0.38 ***	0.58 ***	0.22 *	0.06	0.23 *
LA^*§*^	-0.18	-0.71 ***		-0.07	-0.17	0.09	-0.12	0.79 ***	0.56 ***	-0.26 *	0.26 *	-0.10	-0.07
SLA	-0.16	-0.51 ***	0.18		-0.50 ***	-0.57 ***	-0.50 ***	0.11	-0.03	-0.29 **	0.05	-0.26 *	-0.10
LDMC	-0.70 ***	0.42 **	-0.09	-0.24		0.80 ***	-0.21	-0.19	0.05	0.49 ***	-0.01	0.15	0.18
LD	-0.72 ***	0.49 ***	-0.16	-0.29 *	0.64 ***		-0.27 *	0.07	0.34 **	0.45 ***	0.17	0.08	0.16
LT	0.78 ***	-0.05	-0.01	-0.47 ***	-0.44 **	-0.65 ***		-0.24 *	-0.31 **	-0.08	-0.15	0.23 *	-0.09
PL^*§*^	-0.04	-0.77 ***	0.63 ***	0.09	-0.18	-0.27	0.15		0.57 ***	-0.35 **	0.36 ***	-0.31 **	-0.17
LC	-0.77 ***	-0.10	0.28 *	0.09	0.63 ***	0.40 **	-0.41 **	0.15		-0.07	0.47 ***	-0.18	0.09
WD	-0.41 **	0.59 ***	-0.29 *	-0.26	0.54 ***	0.51 ***	-0.29 *	-0.39 **	0.28 *		-0.11	0.11	0.29 **
De	-0.76 ***	-0.43 **	0.28	0.28	0.28 *	0.30 *	-0.49 ***	0.36 **	0.55 ***	0.13		-0.48 ***	-0.05
Di	0.76 ***	0.43 **	-0.28	-0.28	-0.28 *	-0.30 *	0.49 ***	-0.36 **	-0.55 ***	-0.13	-1.00 ***		0.21
SV	0.50 ***	-0.02	0.25	-0.31 *	-0.30 *	-0.30 *	0.52 ***	0.18	-0.34 *	-0.27	-0.50 ***	0.50 ***	

Relations between the binary variables (LC, De and Di) are Phi coefficients.

^§^Variable was ln-transformed. Lower-left half of the matrix corresponds to dry forest species (n = 51), Upper-right half corresponds to wet forest species (n = 81).

* P < 0.05,

** P < 0.01,

*** P < 0.001.

There were also some differences between forest types. For example, plants with large leaf laminas and petioles (high LA and PL) had an acquisitive strategy in dry forest (as they were associated with high SLA), whereas such plants rather coincided with a drought avoiding strategy in wet forest (as they were associated with deciduousness, Fig 1). Moreover, in dry forest a conservative strategy tended to be associated with a drought avoiding strategy, as the suite of conservative traits (LD, LDMC, WD) tended towards the left side of the biplot where species that are deciduous are positioned. Instead, in wet forest a conservative strategy tended to be associated with species that also have large seeds and that are biotically dispersed, as the conservative traits tended towards the right side of the biplot where evergreen species that invest in large biotically dispersed seeds are positioned.

Phylogenetic analyses showed that most traits were distributed non-randomly over the phylogenetic tree (S2 Table). Correlating the coefficients of the pairwise trait associations (Table 2) with the associations based on their phylogenetic independent contrast (S3 Table) resulted in very tight relationships (Pearson coefficients of 0.97, P< 0.001, for both dry and wet forest), indicating that the phylogenetic signal did not confound the multivariate trait strategies found in this study.

**Fig 3:**
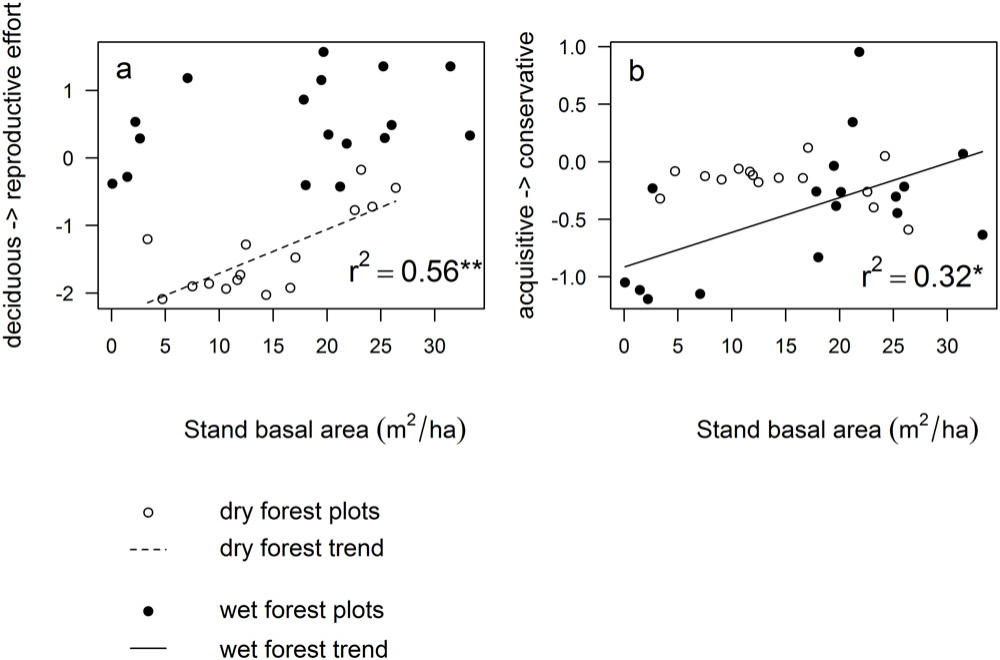
Changes in the dominant plant strategies with succession. Stand basal area was used to indicate succession; it increased asymptotically with successional age and reflects successional change in vegetation structure. Functional composition was calculated using the community-weighted mean of species scores on the principal component axes. (a) Dry forest succession (open symbols, broken regression line) was characterized by changes along the first PCA axis (Fig 1a) and reflected changes from deciduous species to evergreen species that invest in a secure reproductive strategy. (b) Wet forest succession (filled symbols, continuous regression line) was characterized by changes along the second PCA axis (Fig 1b) and reflected changes from an acquisitive strategy to a conservative strategy. Given is the r^2^, * P < 0.05; ** P < 0.01. See S1 Fig for the trends with fallow age as an indicator of succession.

Directional changes in community-weighted PCA scores indicated successional turnover in multivariate plant strategies for both forest types (Fig 3). Interestingly, the main axis that mattered was different for dry and wet forest. Successional changes in dry forest were associated with increasing species scores along the first PCA axis (from high importance of deciduousness early in succession to increased reproductive effort later in succession; Fig 3a), whereas successional changes in wet forest were associated with increasing species scores along the second PCA axis (from acquisitive trait values early in succession to conservative trait values later in succession; Fig 3b). Results were similar when using age instead of basal area, though dry forest change in multivariate plant strategies proved somewhat stronger whereas wet forest change was weaker and no longer significant (see S1 Fig).

## Discussion

We found that tree species from communities growing under very contrasting conditions (dry and wet) face similar functional trait trade-offs, thus confirming the existence of universal trait spectra. The functional turnover with succession in the two forest types, however, reflected different trait spectra, and hence, the changing dominance of different plant strategies. During dry forest succession, species strategies shifted from high importance of deciduousness early in succession towards increased reproductive effort late in succession, whereas during wet forest succession species strategies changed from acquisitive towards conservative strategies. This indicated that dry and wet forest species face different filters during forest succession.

Associations between traits may be influenced by evolutionary history, where the presence of particular clades with contrasting characteristics could confound their ecological interpretation [32]. Phylogenetic analyses showed that although most traits showed significant phylogenetic signal, this did not influence the trait associations found, similar to previous studies, e.g. [6]. Therefore, below we discuss the multivariate trait spectra found in this manuscript in terms of ecological strategies and their relevance for succession in dry and wet tropical forest.

### Dry and wet secondary forest species showed similar trait trade-offs

We hypothesized the existence of two major trade-off axes underlying trait variation in dry and wet forest species, namely the acquisitive-conservative spectrum, and the seed size spectrum, with the spectra reflecting multivariate strategy axes. Our results largely confirmed this hypothesis. The first principal component reflected variation from a deciduous strategy with abiotically (mainly wind-) dispersed species towards evergreen species that invested in biotic seed dispersal, in the dry forest biotic seed dispersal also coincided with an increased seed size (Fig 1). Deciduous species shed their leaves to avoid desiccation and this is an important adaptation to survive severe droughts [17,37,38], which are common in dry forest sites. In both dry and wet forests, deciduous species often also had compound leaves. Compound-leaved species often have photonastic leaves, which can avoid high insolation and therefore high temperature and excessive evaporation by folding their leaflets at noon or during the dry season (e.g., some Fabaceae species). Compoundness also increases leaf cooling and control of water loss [39] and is an efficient way of increasing leaf area for light capture [40]. In both dry and wet forest deciduousness was independent of the acquisitive-conservative continuum, suggesting that deciduous and evergreen species can possess similar resource economics. This is contrary to previous research in temperate forests [41] and across forest types [42]. In line with our results, evidence from another Mexican dry forest shows that the deciduous-evergreen dichotomy does not adequately reflect the variation in leaf and stem functional traits [43]; instead, the *duration* of leaf retention during the dry season reflects this variation better and correlates with resource economics, where conservative species retain their leaves longer during the dry season.

Biotically dispersed, evergreen species, having large seeds (in dry forest) and thick leaves marked the other end of the deciduousness/reproductive effort strategy axis. The positive correlation between seed size and biotic dispersal in dry forest has been widely found [44]. The lack of association in wet forest could be due to the fact that most species are biotically dispersed, here differences in seed volume may instead be related to different animal disperser-groups rather than the abiotic-biotic dichotomy. Biotic dispersal enhances the chance to be dispersed to safe sites, whereas larger seed size increases establishment success [12], which is important in shaded environments [13]. Across plant communities thicker leaves are associated with evergreen plants, confirming leaf thickness as a predictor of leaf lifespan [45]. Within a Bolivian tropical moist forest, however, leaf thickness is largely unrelated to leaf lifespan [46]. The association between abiotic dispersal and deciduousness was expected: wind dispersal is common in tropical dry forest and such wind-dispersed seeds are predominantly dispersed in the dry season, when most deciduous species have shed their leaves and the forest canopy is more open, leading to more efficient wind dispersal [47,48].

The second trade-off axis reflected the strategy axis of resource acquisition versus conservation, in line with the leaf-, stem- and plant economics spectrum, and the growth-survival trade-off [1–3,5,7,49]. Species with cost-efficient leaf area display (high SLA) marked the acquisitive side of this strategy axis; in dry forest this was also associated with large laminas and petioles. High SLA enhances light capture, leaf cooling and gas exchange and enables high photosynthetic capacity and growth rates, e.g. [50]. Species with high leaf density, LDMC and WD marked the conservative side of this strategy axis. Leaf dry matter content and leaf density are indicators of leaf lifespan, resistance against damage [51] and tolerance to drought; dense leaves have smaller cells with thicker and firmer cell walls restricting the modulus of elasticity, thereby avoiding loss of turgor at low leaf water potential [52,53]. High WD is associated with thin and short xylem vessels, thick cell walls, small pit-pores and decreased lumen area, and thus species with dense wood are more resistant against xylem cavitation [54], but see also [55]. High WD also reduces the risk of damage in storms and of stem rot by pathogens [56,57], and indicates drought resistance in drier habitats, where xylem cavitation is the most important cause of tree death [58]. Notably, in our study wood density was associated with the leaf economics spectrum, in line with previous work linking stem and leaf economics [5,43,59], but contrasting with studies suggesting that leaf economics spectrum and wood economics spectrum are largely decoupled [49,60].

### Dry and wet forest succession are characterized by different multivariate strategy axes

We used the community-weighted means of species scores on the two PCA axes to quantify the position of secondary forest communities along these spectra (or multivariate strategy axes) of trait variation. Doing so, we found that in both dry and wet forest, directional changes in the dominance of plant strategies took place with secondary succession (Fig 3). However, the main axis of change was different for dry and wet forest. We found that the first PCA axis, reflecting seed size and deciduousness, was the main axis for successional change in dry forest while the second PCA axis, reflecting the acquisitive-conservative strategy axis, was the main axis for successional change in wet forest (Fig 3). This indicated that successional changes in multivariate plant strategies in dry and wet tropical forest were characterized by independent axes of plant strategy variation. If indeed dry forest succession is mainly driven by the water gradient and wet forest succession by the light gradient, this would indicate that drought and shade tolerance are largely decoupled, and that these abilities depend on different trait combinations, as has been found in other studies [61–63].

In dry forest the main axis of variation was not the acquisitive conservative trade-off, as we anticipated, but the axis that described seed size and drought avoidance strategies. Dry forest changes in functional composition were characterized by the gradient of compound- leaved, deciduous species early in succession towards larger-seeded species that were more often animal dispersed and had thicker leaves later in succession. This finding confirms previous studies showing that deciduousness and leaf compoundness are particularly important during the extra dry environments in early-successional stages, cf. [17,37,64]. The proportion of species that depend on animals for seed dispersal increased during tropical dry forest succession (though it remained low compared to wet forest sites: Fig 3a), as did the seed size. This confirms that early-successional species invest in many small seeds that can travel large distances (e.g., by wind), whereas late-successional species are more likely to invest in fruits that attract biotic dispersers to enhance directional dispersal. Given that the second principal component (acquisitive/conservative strategy axis) was relatively unimportant, it is likely that in our dry forest sequence drought avoidance (characterized by deciduousness) was more important than drought resistance (characterized by conservative traits).

In wet forest, the main axis of variation was described by changes in functional composition from acquisitive to conservative trait values (Fig 3b), a result that complies with expectations based on decreasing light availability during succession [18,24,65–67]. Regenerative traits did not play a role in species assembly along the gradient of wet forest succession as we found no increase in biotically-dispersed trees, nor an increase in seed size. Instead, biotic dispersal was common throughout the successional gradient, in line with previous studies [68]. Increasing seed size, an important trait for establishment success under shaded conditions [13] was not found; possibly it could start playing a role at later successional stages or in forest positioned in a more intact landscape forest-matrix.

We investigated a dry (900 mm/yr) and a wet forest (3000 mm/yr) chronosequence and showed that tree species are constrained by similar trade-offs, though this had different consequences for the success of plant strategies during succession. This confirms that dry and wet forest species face different filters during succession. A challenging issue is how the relative strength of these different filters (light and water) changes along the large precipitation gradient found across tropical regions and the consequences thereof for functional composition of successional communities. This is relevant because throughout the tropics the importance of secondary and degraded forests is increasing [69] and there is great need to understand its effects on biodiversity and ecosystem functioning [70]. Moreover, restoration plantings with local species that mimic natural regeneration may be needed to speed up forest recovery and improve biodiversity conservation and ecosystem services delivery [71]. A switch from water being replaced by light as the main filter somewhere along the precipitation gradient has direct consequences for forest restoration activities and the selection of to-be-planted species with characteristics that fit with the main filters, cf. [72].

This study showed that similar trait spectra were observed among dry and wet secondary forest species, but with different consequences for succession. In dry forest succession the dominant plant strategies changed from drought avoiding species towards species that invest in large biotically dispersed seeds, which can be explained by water limitations in early succession. In wet forest succession the dominant plant strategies changed from species having acquisitive towards species with conservative strategies, which can be explained by decreasing light availability as the main driver of wet forest succession.

## Supporting Information

S1 Dataset This file contains data belonging to the article " Functional trait strategies of trees in dry and wet tropical forests are similar but differ in their consequences for succession" by M. Lohbeck, E. Lebrija-Trejos, M. Martínez-Ramos, J.A. Meave, L. Poorter and F. Bongers. Data are presented per forest type, the first two sheets containing the data from the Principal Components Analyses (Fig 1). Presented are the traits, their eigenvector scores and the species scores on the first four axes. The last two sheets present the secondary forest plot data, their fallow ages, stand basal area and their Community-Weighted Mean scores on the first two PCA axes (see methods, Fig 3 and S1 Fig).
(XLSX)

S1 Fig Changes in the dominant plant strategies with succession, using two different indicators of succession: stand basal area (a, b) and fallow age (c, d). Functional composition was calculated using the community-weighted mean of species scores on the principal component axes (Fig 1). Dry forest succession (open symbols, [d], broken regression line) was characterized by changes along the first PCA axis and reflected changes from deciduous species to evergreen species that invest in a secure reproductive strategy. This was significant when using stand basal area as a successional indicator (a), and when using fallow age (c). Wet forest succession (solid symbols, [w], continuous regression line) was characterized by changes along the second PCA axis and reflected changes from an acquisitive strategy to a conservative strategy. This was significant when using stand basal area as successional indicator (b), but not when using fallow age (d). Given is the r^2^, * P < 0.05; ** P < 0.01.
(TIFF)

S1 Table List of species included in this study, in alphabetical order and grouped per forest type. These species represent at least 80% of the basal area of each secondary forest plot. All species except Aragebortia sp. (wet forest) were used in the phylogenetic analysis, as for this species the family was unknown.
(DOCX)

S2 Table Phylogenetic signal for each of the functional traits for the two forest types (a: dry forest, b: wet forest). Given are Blomberg’s K [29], the variance based on the observed trait distribution on the phylogeny, the randomized mean and the statistical significance of the difference between the observed phylogenetic signal and the random scenario (based on 999 randomizations).
(DOCX)

S3 Table Spearman coefficients of the pairwise relations between Phylogenetic Independent Contrasts. Relations between the binary variables (LC, De and Di) are Phi coefficients. ^§^Traits were ln-transformed prior to PIC calculation. Lower-left half of the matrix corresponds to dry forest species (n = 51), Upper-right half corresponds to wet forest species (n = 80). * P < 0.05, ** P < 0.01, *** P < 0.001. These values are very similar to the original pairwise trait-correlations (Table 2), as resulting from the strong correlation between the correlation coefficient in this table and those of Table 2 (Pearson 0.97, P< 0.001)
(DOCX)
