# Supplementary figures and images for: Functional Trait Strategies of Trees in Dry and Wet Tropical Forests Are Similar but Differ in Their Consequences for Succession
Source: PLoS One. 2015 Apr 28;10(4):e0123741. doi: 10.1371/journal.pone.0123741 (PMC4412708; doi:10.1371/journal.pone.0123741)

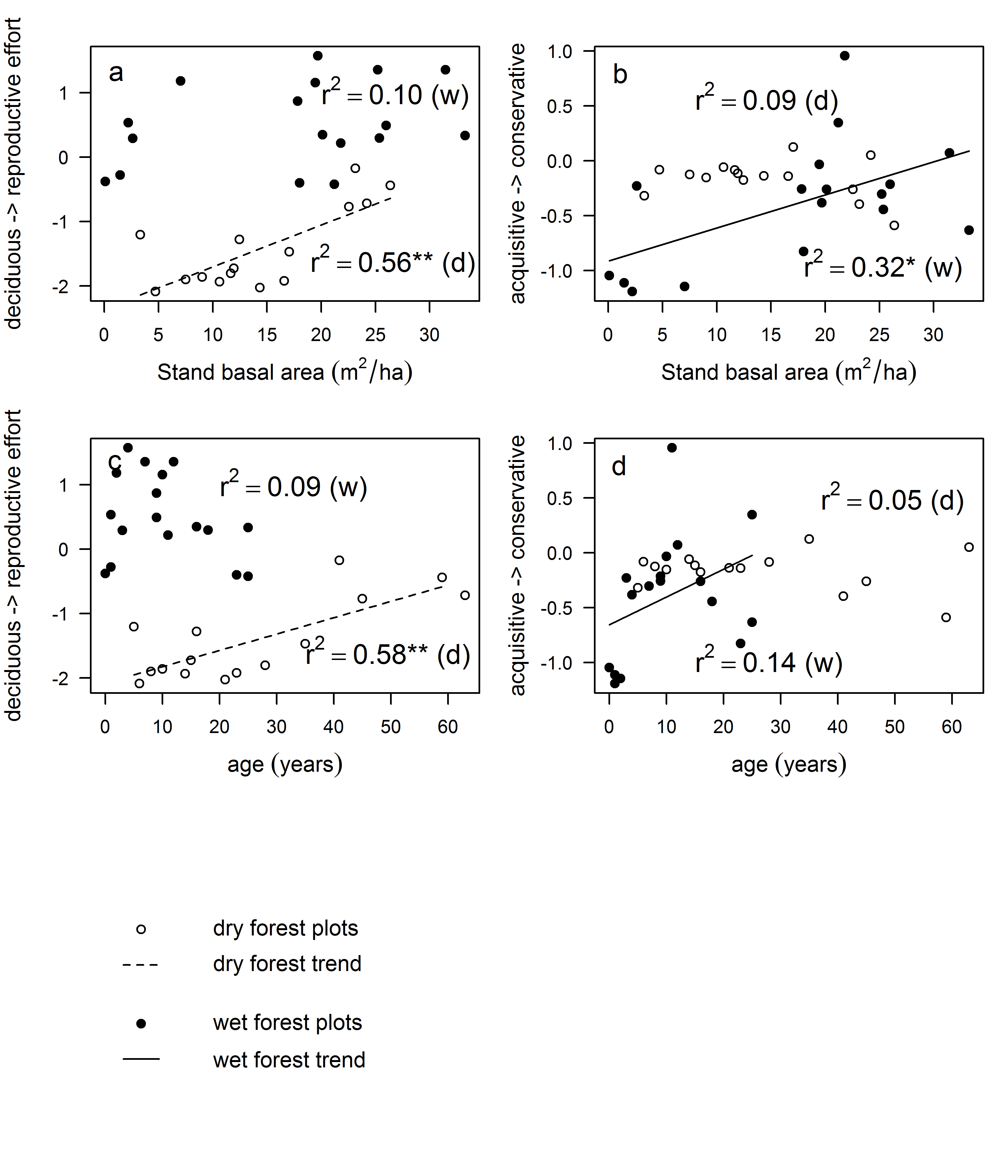

Supplement: S1 Fig — Functional composition was calculated using the community-weighted mean of species scores on the principal component axes (Fig 1). Dry forest succession (open symbols, [d], broken regression line) was characterized by changes along the first PCA axis and reflected changes from deciduous species to evergreen species that invest in a secure reproductive strategy. This was significant when using stand basal area as a successional indicator (a), and when using fallow age (c). Wet forest succession (solid symbols, [w], continuous regression line) was characterized by changes along the second PCA axis and reflected changes from an acquisitive strategy to a conservative strategy. This was significant when using stand basal area as successional indicator (b), but not when using fallow age (d). Given is the r2, * P < 0.05; ** P < 0.01. (TIFF) [file pone.0123741.s002.tiff]
