# Supplementary material for: Functional Trait Strategies of Trees in Dry and Wet Tropical Forests Are Similar but Differ in Their Consequences for Succession
Source: PLoS One. 2015 Apr 28;10(4):e0123741. doi: 10.1371/journal.pone.0123741 (PMC4412708; doi:10.1371/journal.pone.0123741)
Supplement: S1 Table — These species represent at least 80% of the basal area of each secondary forest plot. All species except Aragebortia sp. (wet forest) were used in the phylogenetic analysis, as for this species the family was unknown. (DOCX) [file pone.0123741.s003.docx]

Dry forest species:

Fabaceae/Acacia/Acacia_cochliacantha

Fabaceae/Acacia/Acacia_farnesiana

Fabaceae/Acacia/Acacia_picachensis

Fabaceae/Aeschynomene/Aeschynomene_compacta

Anacardiaceae/Amphipterygium/Amphipterygium_adstringens

Fabaceae/Apoplanesia/Apoplanesia_paniculata

Bignoniaceae/Arrabidaea/Arrabidaea_costaricensis

Burseraceae/Bursera/Bursera_excelsa

Burseraceae/Bursera/Bursera_simaruba

Fabaceae/Caesalpinia/Caesalpinia_platyloba

Capparaceae/Capparis/Capparis_incana

Salicaceae/Casearia/Casearia_tremula

Malvaceae/Ceiba/Ceiba_parvifolia

Fabaceae/Chloroleucon/Chloroleucon_mangense

Euphorbiaceae/Cnidoscolus/Cnidoscolus_megacanthus

Polygonaceae/Coccoloba/Coccoloba_liebmannii

Boraginaceae/Cordia/Cordia_dentata

Euphorbiaceae/Croton/Croton_niveus

Euphorbiaceae/Croton/Croton_pseudoniveus

Euphorbiaceae/Euphorbia/Euphorbia_schlechtendalii

Rubiaceae/Exostema/Exostema_caribaeum

Resedaceae/Forchhammeria/Forchhammeria_pallida

Hernandiaceae/Gyrocarpus/Gyrocarpus_mocinnoi

Fabaceae/Havardia/Havardia_campylacantha

Malvaceae/Heliocarpus/Heliocarpus_pallidus

Fabaceae/Indigofera/Indigofera_thibaudiana

Primulaceae/Jacquinia/Jacquinia_macrocarcarpa

Euphorbiaceae/Jatropha/Jatropha_alamanii

Rhamnaceae/Krugiodendron/Krugiodendron_ferreum

Fabaceae/Leucaena/Leucaena_lanceolata

Fabaceae/Lonchocarpus/Lonchocarpus_lanceolatus

Fabaceae/Lonchocarpus/Lonchocarpus_torresiorum

Fabaceae/Lysiloma/Lysiloma_divaricatum

Malpighiaceae/Malpighia/Malpighia_emarginata

Euphorbiaceae/Manihot/Manihot_oaxacana

Fabaceae/Mimosa eurycarpa/Mimosa_eurycarpa

Fabaceae/Mimosa/Mimosa_goldmanii

Fabaceae/Mimosa/Mimosa_tenuiflora

Fabaceae/Myrospermum/Myrospermum_frutescens

Fabaceae/Piptadenia/Piptadenia_flava

Fabaceae/Piptadenia/Piptadenia_obliqua

Rubiaceae/Randia/Randia_thurberi

Schoepfiaceae/Schoepfia/Schoepfia_schreberi

Fabaceae/Senna/Senna_atomaria

Fabaceae/Senna/Senna_holwayana

Apocynaceae/Tabernaemontana/Tabernaemontana_glabra

Bignoniaceae/Tabebuia/Tabebuia_impetiginosa

Apocynaceae/Thevetia/Thevetia_plumeriifolia

Sapindaceae/Thouinia/Thouinia_villosa

Sapindaceae/Thouinidium/Thouinidium_decandrum

Rutaceae/Zanthoxylum/Zanthoxylum_caribaeum

Wet forest species:

Verbenaceae/Aegiphila/Aegiphila_monstrosa

Euphorbiaceae/Alchornea/Alchornea_latifolia

Rubiaceae/Alibertia/Alibertia_edulis

Ulmaceae/Ampelocera/Ampelocera_hottlei

Bignoniaceae/Amphitecna/Amphitecna_apiculata

?/Aragebortia/Aragebortia_sp

Anacardiaceae/Astronium/Astronium_graveolens

Melastomataceae/Bellucia/Bellucia_axinanthera

Rubiaceae/Blepharidium/Blepharidium_mexicanum

Moraceae/Brosimum/Brosimum_alicastrum

Moraceae/Brosimum/Brosimum_guianensis

Burseraceae/Bursera/Bursera_simaruba

Clusiaceae/Calophyllum/Calophyllum_brasiliense

Salicaceae/Casearia/Casearia_sylvestris

Urticaceae/Cecropia/Cecropia_peltata

Malvaceae/Ceiba/Ceiba_pentandra

Melastomataceae/Conostegia/Conostegia_xalapensis

Boraginaceae/Cordia/Cordia_alliodora

Boraginaceae/Cordia/Cordia_diversifolia

Euphorbiaceae/Croton/Croton_schiedeanus

Sapindaceae/Cupania/Cupania_dentata

Sapindaceae/Cupania glabra/Cupania_glabra

Fabaceae/Dalbergia/Dalbergia_glabra

Araliaceae/Dendropanax/Dendropanax_arboreus

Fabaceae/Dialium/Dialium_guianense

Fabaceae/Erythrina/Erythrina_folkersii

Myrtaceae/Eugenia/Eugenia_acapulcensis

Myrtaceae/Eugenia/Eugenia_nigrita

Rubiaceae/Faramea/Faramea_occidentalis

Fabaceae/Gliricidia/Gliricidia_sepium

Meliaceae/GuareA/Guarea_glabra_

Rubiaceae/Hamelia/Hamelia_patens

Malvaceae/Heliocarpus/Heliocarpus_appendiculatus

Chrysobalanaceae/Hirtella/Hirtella_americana

Chrysobalanaceae/Hirtella/Hirtella_racemosa

Fabaceae/Inga/Inga_pavoniana

Amaranthaceae/Iresine/Iresine_arbuscula

Lacistemataceae/Lacistema/Lacistema_aggregatum

Chrysobalanaceae/Licania/Licania_hypoleuca

Chrysobalanaceae/Licania/Licania_platypus

Fabaceae/Indigofera/Indigofera_frutescens

Malvaceae/Luehea/Luehea_speciosa

Melastomataceae/Miconia/Miconia_ampia

Melastomataceae/Miconia/Miconia_glaberrima

Melastomataceae/Miconia/Miconia_trinervia

Lauraceae/Nectandra/Nectandra_reticulata

Lauraceae/Nectandra/Nectandra_salicifolia

Malvaceae/Ochroma/Ochroma_pyramidale

Araliaceae/Oreopanax/Oreopanax_peltatus

Primulaceae/Parathesis/Parathesis_lenticellata

Ulmaceae/Phylostilum/Phylostilum_subsecile

Piperaceae/Piper/Piper_aduncum

Piperaceae/Piper /Piper_hispidum

Piperaceae/Piper/Piper_auritum

Piperaceae/Piper/Piper_sanguinea

Rubiaceae/Posoqueria/Posoqueria_latifolia

Sapotaceae/Pouteria/Pouteria_durlandii

Burseraceae/Protium/Protium_copal

Myrtaceae/Psidium/Psidium_friedrichsthalianum

Fabaceae/Schizolobium/Schizolobium_parahybum

Fabaceae/Senna papillosa/Senna_papillosa

Sapotaceae/Sideroxylon/Sideroxylon_nigra

Monimiaceae/Siparuna/Siparuna_andina

Solanaceae/Solanum/Solanum_rudepanum

Solanaceae/Solanum umbelatum/Solanum_umbelatum

Anacardiaceae/Spondias radlkoferi/Spondias_radlkoferi

Apocynaceae/Tabernaemontana/Tabernaemontana_alba

Combretaceae/Terminalia/Terminalia_amazonia

Apocynaceae/Thevetia/Thevetia_ahouai

Ulmaceae/Trema/Trema_laxiflora

Ulmaceae/Trema/Trema_micrantha

Malvaceae/Trichospermum/Trichospermum_mexicanum

Fabaceae/Vatairea/Vatairea_lundellii

Asteraceae/Vernonia/Vernonia_patens

Clusiaceae/Vismia/Vismia_camparaguey

Vochysiaceae/Vochysia/Vochysia_guatemalensis

Annonaceae/Xylopia/Xylopia_frutescens

Rutaceae/Zanthoxylum/Zanthoxylum_caribaeum

Rutaceae/Zanthoxylum/Zanthoxylum_kellermanii

Rutaceae/Zanthoxylum/Zanthoxylum_procerum

Salicaceae/Zuelania/Zuelania_guidonia
