# Supplementary material for: Functional Trait Strategies of Trees in Dry and Wet Tropical Forests Are Similar but Differ in Their Consequences for Succession
Source: PLoS One. 2015 Apr 28;10(4):e0123741. doi: 10.1371/journal.pone.0123741 (PMC4412708; doi:10.1371/journal.pone.0123741)
Supplement: S2 Table — Given are Blomberg’s K [29], the variance based on the observed trait distribution on the phylogeny, the randomized mean and the statistical significance of the difference between the observed phylogenetic signal and the random scenario (based on 999 randomizations). (DOCX) [file pone.0123741.s004.docx]

| a. Dry forest | K | PIC.variance.obs | PIC.variance.rnd.mean | PIC.variance.P |
| --- | --- | --- | --- | --- |
| De | 0.35 | 6.17E-02 | 1.04E-01 | 0.001 |
| Di | 0.35 | 6.17E-02 | 1.05E-01 | 0.003 |
| LC | 1.20 | 2.42E-02 | 1.17E-01 | 0.001 |
| SLA | 0.20 | 7.70E+00 | 7.39E+00 | 0.592 |
| LDMC | 0.63 | 2.03E-03 | 5.26E-03 | 0.001 |
| LD | 0.26 | 1.42E-02 | 1.53E-02 | 0.328 |
| LT | 0.41 | 1.23E-03 | 2.15E-03 | 0.003 |
| WD | 0.59 | 7.11E-03 | 1.74E-02 | 0.001 |
| SV | 0.16 | 5.43E+05 | 4.18E+05 | 0.735 |
| LA | 0.27 | 7.03E-02 | 9.17E-02 | 0.065 |
| PL | 0.41 | 4.09E-02 | 8.21E-02 | 0.001 |
| b. Wet forest | | | | |
| De | 0.25 | 4.20E-02 | 4.99E-02 | 0.138 |
| Di | 0.49 | 3.97E-02 | 9.32E-02 | 0.001 |
| LC | 0.95 | 2.36E-02 | 1.06E-01 | 0.001 |
| SLA | 0.29 | 6.65E+00 | 9.13E+00 | 0.008 |
| LDMC | 0.34 | 2.62E-03 | 4.24E-03 | 0.001 |
| LD | 0.36 | 5.48E-03 | 9.20E-03 | 0.001 |
| LT | 0.26 | 1.55E-03 | 1.88E-03 | 0.175 |
| WD | 0.32 | 1.06E-02 | 1.63E-02 | 0.001 |
| SV | 0.18 | 4.46E+06 | 3.79E+06 | 0.766 |
| LA | 0.37 | 1.01E-01 | 1.74E-01 | 0.001 |
| PL | 0.33 | 9.83E-02 | 1.49E-01 | 0.002 |
