# Supplementary material for: Functional Trait Strategies of Trees in Dry and Wet Tropical Forests Are Similar but Differ in Their Consequences for Succession
Source: PLoS One. 2015 Apr 28;10(4):e0123741. doi: 10.1371/journal.pone.0123741 (PMC4412708; doi:10.1371/journal.pone.0123741)
Supplement: S3 Table — Relations between the binary variables (LC, De and Di) are Phi coefficients. §Traits were ln-transformed prior to PIC calculation. Lower-left half of the matrix corresponds to dry forest species (n = 51), Upper-right half corresponds to wet forest species (n = 80). * P < 0.05, ** P < 0.01, *** P < 0.001. These values are very similar to the original pairwise trait-correlations (Table 2), as resulting from the strong correlation between the correlation coefficient in this table and those of Table 2 (Pearson 0.97, P< 0.001) (DOCX) [file pone.0123741.s005.docx]

|  | LA^§^ | SLA | LDMC | LD | LT | PL^§^ | LC | WD | De | Di | SV |
| --- | --- | --- | --- | --- | --- | --- | --- | --- | --- | --- | --- |
| LA^§^ |  | 0.04 | -0.33 ** | -0.08 | -0.23 * | 0.80 *** | 0.54 *** | -0.33 ** | 0.22 | -0.14 | 0.05 |
| SLA | 0.22 |  | -0.42 *** | -0.48 *** | -0.49 *** | 0.24* | -0.02 | -0.37 ** | -0.11 | -0.07 | -0.17 |
| LDMC | -0.22 | -0.36 * |  | 0.77*** | -0.16 | -0.29 ** | 0.07 | 0.48 *** | 0.02 | 0.12 | 0.10 |
| LD | -0.15 | -0.26 | 0.53 *** |  | -0.32** | 0.00 | 0.28 * | 0.46 *** | 0.17 | -0.02 | 0.13 |
| LT | 0.03 | -0.36* | -0.36 * | -0.70 *** |  | -0.38 *** | -0.35 ** | -0.05 | -0.11 | 0.11 | -0.02 |
| PL^§^ | 0.55 *** | 0.13 | -0.23 | -0.29* | 0.11 |  | 0.57 *** | -0.41 *** | 0.30 ** | -0.28 * | -0.10 |
| LC | 0.19 | -0.15 | 0.59 *** | 0.45 ** | -0.38 ** | 0.13 |  | -0.13 | 0.51 *** | -0.28 * | -0.01 |
| WD | -0.26 | -0.39 ** | 0.62 *** | 0.49*** | -0.26 | -0.44 ** | 0.24 |  | 0.01 | 0.12 | 0.25 * |
| De | 0.23 | 0.14 | 0.21 | 0.32* | -0.44 ** | 0.33 * | 0.56 *** | 0.09 |  | -0.67 *** | -0.14 |
| Di | -0.23 | -0.14 | -0.21 | -0.32 * | 0.44 ** | -0.33 * | -0.56 *** | -0.09 | -1 *** |  | 0.10 |
| SV | 0.19 | 0.01 | -0.49 *** | -0.29 * | 0.40 ** | 0.23 | -0.33 * | -0.42 ** | -0.48 *** | 0.48 *** |  |
